# Associating Type 2 Diabetes Risk Factor Genes and FDG-PET Brain Metabolism in Normal Aging and Alzheimer’s Disease

**DOI:** 10.3389/fnagi.2020.580633

**Published:** 2020-10-30

**Authors:** Scott Nugent, Olivier Potvin, Stephen C. Cunnane, Ting-Huei Chen, Simon Duchesne

**Affiliations:** ^1^Centre de Recherche CERVO de l’Institut Universitaire en Santé Mentale de Québec, Québec, QC, Canada; ^2^Research Center on Aging, Health and Social Sciences Center, Geriatrics Institute, Sherbrooke, QC, Canada; ^3^Département de Mathématiques et Statistiques, Faculté des Sciences et de génie, Université Laval, Québec, QC, Canada; ^4^Département de Radiologie et Médecine Nucléaire, Faculté de Médecine, Université Laval, Québec, QC, Canada

**Keywords:** normal aging brain, Alzhermer disease, type 2 diabetes, gene expression, brain metabolic imaging

## Abstract

**Background**: Several studies have linked type 2 diabetes (T2D) to an increased risk of developing Alzheimer’s disease (AD). This has led to an interest in using antidiabetic treatments for the prevention of AD. However, the underlying mechanisms explaining the relationship between T2D and AD have not been completely elucidated.

**Objective**: Our objective was to examine cerebral ^18^F-fluorodeoxyglucose (FDG) uptake during normal aging and in AD patients in regions associated with diabetes genetic risk factor expression to highlight which genes may serve as potential targets for pharmaceutical intervention.

**Methods**: We calculated regional glucose metabolism differences in units of standardized uptake values (SUVR) for 386 cognitively healthy adults and 335 clinically probable AD patients. We then proceeded to extract gene-expression data from the publicly available Allen Human Brain Atlas (HBA) database. We used the nearest genes to 46 AD- and T2D-associated SNPs previously identified in the literature, and mapped their expression to the same 34 cortical regions in which we calculated SUVRs. SNPs with a donor consistency of 0.40 or greater were selected for further analysis. We evaluated the associations between SUVR and gene-expression across the brain.

**Results**: Of the 46 risk-factor genes, 15 were found to be significantly correlated with FDG-PET brain metabolism in healthy adults and probable AD patients after correction for multiple comparisons. Using multiple regression, we found that five genes explained a total of 72.5% of the SUVR variance across the healthy adult group regions, while four genes explained a total of 79.3% of the SUVR variance across the probable AD group regions. There were significant differences in whole-brain SUVR as a function of allele frequencies for two genes.

**Conclusions**: These results highlight the association between risk factor genes for T2D and regional glucose metabolism during both normal aging and in probable AD. Highlighted genes were associated with mitochondrial stability, vascular maintenance, and glucose intolerance. Pharmacological intervention of these pathways has the potential to improve glucose metabolism during normal again as well as in AD patients.

## Introduction

There is a considerable body of literature on associations between type 2 diabetes (T2D) and dementia, especially Alzheimer’s disease (AD). A 2009 meta-analysis by Lu et al. (2009) calculated that individuals with T2D were 39% more likely to develop AD than non-diabetics; reports since then have confirmed this finding. In a review, Strachan et al. (2011) reported that T2D led to a 1.5 to 2.5-fold increase in the risk for all-cause dementia. Cheng et al. (2012) found that participants with T2D had a 21% higher risk for mild cognitive impairment (MCI), 46% higher risk for AD, and 51% higher risk for any dementia than participants without diabetes.

This association has led to an increased interest in using anti-diabetic treatment for the prevention of AD. Li et al. (2015) reviewed the efficacy of anti-diabetic treatment in AD clinical trials and found that several of the current anti-diabetic medication was effective at delaying the symptoms or onset of AD. However, a Cochrane review did not find any effects of diabetic conventional treatments in preventing cognitive impairment in individuals with T2D (Areosa Sastre et al., 2017). The authors acknowledged that longer follow-up studies are necessary to assess the impact of T2D on the progression from MCI to AD.

The association between T2D and AD is correlated to changes in dementia biomarkers. For example, Schneider et al. (2007) studied the association between T2D, brain volumes, and vascular pathology. The authors found that T2D participants with high levels of glycated hemoglobin (HbA1C) had smaller brain volumes across the whole cortex and a higher burden of lacunes.

Baker et al. (2011) measured ^18^F-fluorodeoxyglucose (FDG) uptake using positron emission tomography (PET) in patients with T2D. Brain glucose metabolism was expressed as standardized uptake values, normalized using the whole brain (SUVR). The authors found that T2D patients had significantly lower SUVR when compared to healthy adults, in regions that are typically hypometabolic in AD patients. In a separate study, Craft et al. (2012) measured FDG-PET uptake following an intranasal insulin therapy in patients with MCI and AD. They found that brain FDG-PET uptake was higher in patients receiving the intervention when compared with placebo.

However, pathophysiologically the relationship has not been completely elucidated. de La Monte (2017) has suggested that much of the mechanisms involved in AD can be attributed to impairments in insulin and IGF signaling. Indeed, insulin resistance in T2D has been shown to exacerbate directly amyloid and tau pathologies, and their shared pathophysiological traits of synaptic dysfunction, inflammation, and autophagic impairments (Chatterjee and Mudher, 2018). Further investigations are required to better our understanding of these shared pathways.

One avenue of research is related to the shared genetic components between T2D and AD, which have been reported as possible common mechanisms for disease development (Chatterjee and Mudher, 2018). There have been limited studies however on the functionality of these shared genetic components.

In a recent study, our group examined 40 T2D risk-factor single-nucleotide polymorphisms (SNPs) and their association with progression from MCI to clinically probable AD (Girard et al., 2018). We found that SNPs located in two genes (SRR and TCF7L2) were most strongly associated with the conversion of MCI to probable AD.

We were interested in furthering this research by examining associations between the *expression* of identified diabetes genetic risk factors and cerebral glucose metabolism using FDG-PET. While genotyping data is profusely available in the literature, localized (voxel-wise) genetic expression across the brain is not. Only one major atlas has been derived and released for such a purpose, namely the Allen Human Brain Atlas (HBA; Allen Institute for Brain Science; Hawrylycz et al., 2012)^1^. A previous report has correlated HBA cell-specific gene expression with regional profiles of MRI-based estimates of cortical thickness (Shin et al., 2018). We wanted to perform a similar experiment towards studying the impact of T2D genetic risk on brain metabolism.

To this end, we analyzed glucose metabolism data from cognitively healthy adults as well as clinically probable AD patients and compared SUVR on a regional basis to gene expression measured in the independent HBA sample. This was done under the strict assumption that gene expression from the HBA would resemble those from our study group. We hypothesized that average regional expression in some genetic markers would be correlated with FDG-PET uptake values. These highlighted genes could then be used as targets for pharmaceutical intervention for improving cerebral glucose metabolism during normal aging and in AD.

## Materials and Methods

### Overview

**Figure 1:**
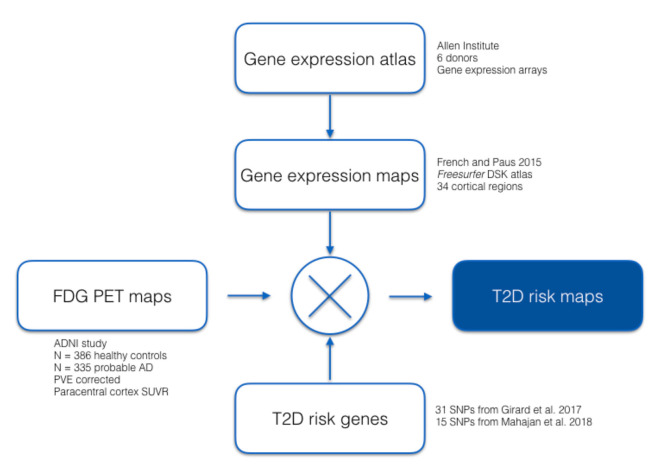
Flowchart describing the datasets and proposed analysis.

We analyzed gene-expression data from the publicly available Allen HBA database (Hawrylycz et al., 2012), which have been mapped by (French and Paus, 2015) from the HBA to the 34 cortical regions defined by the Desikan–Killiany Atlas used by FreeSurfer (Desikan et al., 2006). We then computed regional cerebral glucose metabolism FDG-PET standardized uptake values for participants in the Alzheimer’s Disease Neuroimaging Initiative (ADNI) in the same cortical areas. Using 31 T2D associated SNPs previously identified in our Girard et al.’s (2018) study, as well as 15 T2D associated SNPs validated by Mahajan et al. (2018), we evaluated associations between SUVR and gene-expression across the brain. A summary of our analysis is shown in Figure 1.

### Allen Human Brain Atlas

Gene-expression data were obtained in postmortem human brains from the Allen HBA that provides comprehensive coverage of the normal adult brain (Hawrylycz et al., 2012). Fresh-frozen brains were slabbed, sectioned, and blocked. Expert macrodissection of anatomically defined cortical regions was guided by histological information (Nissl and other markers). Isolated RNA was hybridized to custom 64K Agilent microarrays (58,692 probes). The above steps were carried out by the Allen Institute, which released expression data for the left hemispheres of six donors (five males aged 24, 31, 39, 55, and 57 years; and one female aged 49 years). All donors were free of drugs prescribed for psychiatric disorders.

### Allen HBA Mapping to the Desikan–Killiany Atlas

Using procedures developed by French and Paus (2015), gene-expression data from the Allen HBA was mapped to the 34 left hemisphere cortical regions defined by the Desikan–Killiany Atlas (Desikan et al., 2006).

### FDG-PET ADNI Participant Inclusion Criteria

Participants’ data used in the preparation of this article were obtained from the Alzheimer’s disease Neuroimaging Initiative (ADNI) database^2^ in March 2017. ADNI was launched in 2003 as a public-private partnership, led by Michael W. Weiner^3^. Approval from the local ethics board and informed consent of the participants was obtained as part of the ADNI study.

We included all participants enrolled in the ADNI study that had undergone FDG-PET scanning. Participants were classified as cognitively healthy (CH) or clinically probable AD (Table 1). Probable AD was diagnosed according to the National Institute of Neurological and Communicative Disorders and Stroke and the Alzheimer’s Disease and Related Disorders Association (Mckhann et al., 1984) criteria, with a Clinical Dementia Rating of 1.

**Table 1:** Characteristics of study participants.

	Healthy adults		Alzheimer’s		
	(*n* = 386)		(*n* = 335)		
	Mean	SD	Mean	SD	*p*-value
Age (years)	74.5	6.2	75.5	7.6	0.05
Sex (% Female)	51.5	-	41.2	-	0.02
MMSE	29.0	1.9	23.0	3.4	<0.001
Body mass index (kg/m^2^)	27.4	4.8	25.9	4.8	<0.001
Scanner resolution	5.9	0.7	6.0	0.7	0.14

### FDG-PET Analysis

The FDG-PET processing steps have been described in detail previously (Nugent et al., 2020). In general, steps include co-registration to the first FDG-PET frame; averaging of all timeframes; and partial volume correction using region-based voxel-wise correction, an extension of the geometric transfer matrix method. Partial volume correction was implemented using PETPVC, which is available on GitHub^4^ (further details may be found at Thomas et al., 2011). All PET images were then converted to SUVR by voxel-wise division to the average activity of the pons, which has been validated as the most appropriate reference region for aging research (Nugent et al., 2020). Finally, average SUVR activity was extracted using *FreeSurfer* ROI.

### Selected SNPs

We investigated 31 T2D associated SNPs previously identified in Girard et al. (2018) in addition to 15 T2D associated SNPs validated by Mahajan et al. (2018). The full list of SNPs is summarized in Supplementary Table 1. Sequencing data was sourced from ADNI GO/2 GWAS, derived using an Illumina HumanOmniExpress BeadChip. SNPs with a donor consistency of 0.40 or greater were selected for further analysis to control for the effects of age and sex. Donor consistency was calculated as the average donor correlation to the median for each SNP and provides a measure of consistency across the participant’s brains sampled.

### Statistical Analyses

First, Pearson correlations were used to study associations between ADNI-based SUVR and HBA-based gene expression, with a Bonferroni correction applied for multiple comparisons. Second, to assess the independent association of gene expression and SUVR across regions, we used multiple regression models in the healthy adult and AD groups to predict regional SUVR using regional gene expression as predictors. Models were conducted using the Scikit-learn python module (Pedregosa et al., 2011). All 46 risk-factor genes were entered as predictors and the best ones were selected according to the mean squared error loss using recursive feature elimination with 10-fold cross-validation (RFECV function). *R*^2^ was computed with the r2_score metric function based on 10-fold cross-validation. With the R package relaimpo, the function calc.relimp was used to calculate the relative importance of each predictor that was retained in the model. The lmg metric was used, based on (Lindeman et al., 1980), which calculates an R^2^ partitioned by averaging sequential sums of squares while taking into account all possible orders of the predictors.

## Results

### Sample Characteristics

Table 1 shows the characteristics of the study participants. There were significant differences between healthy adult and AD groups. The AD group was significantly older, had a smaller ratio of females, had lower MMSE, and lower body mass index.

### Linear Regression Models

In the healthy adult group, five genes were retained by the model and explained a total of 72.5% of the SUVR variance across regions. These included TOMM40 (41.4%), PPAP2B (17.9%), PHACTR1 (8.1%), PAM (3.8%) and CDKAL1 (1.2%).

In the probable AD group, four genes explained a total of 79.3% of the SUVR variance across regions. These included TOMM40 (32.0%), ANKH (27.5%), PPAP2B (16.3%), and CDKAL1 (3.56%).

### SUVR—Genetic Expression Correlations

**Figure 2:**
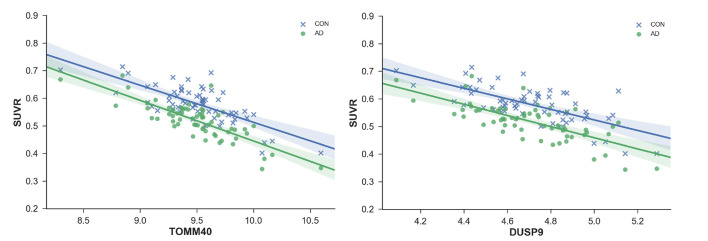
Standardized values (SUVR) plotted against regional gene expression for genes with most significant correlations; TOMM40 and ANKH. Each point represents a region of the Desikan–Killiany (DKT) Atlas.

Of the 46 risk-factor genes, 15 were found to be significantly correlated with FDG-PET brain metabolism in healthy adults and probable AD patients (Table 2). TOMM40 (Figure 2) had the most significant correlation with SUVR (CH: *r* = −0.74, *p* = 8.38E-12; probable AD: *r* = −0.80, *p* = 5.77E-15), followed by ANKH (CH: *r* = 0.69, *p* = 4.2E-10; probable AD: *r* = 0.77, *p* = 2.58E-13), DUSP9 (CH: *r* = −0.69, *p* = 6.05E-10; probable AD: *r* = −0.72, *p* = 3.19E-11), MRPS30 (CH: *r* = 0.65, *p* = 8.15E-09; probable AD: *r* = 0.62, *p* = 7.58E-08), MRAS (CH: *r* = −0.64, *p* = 2.53E-08; probable AD: *r* = −0.70, *p* = 1.84E-10), and LPA (CH: *r* = −0.60, *p* = 1.97E-07; probable AD: *r* = −0.58, *p = 8.38E-07*).

**Table 2:** Correlation between type 2 diabetes risk genes and brain standardized values (SUVR) uptake.

	Healthy adults		AD patients	
	*r*	*p*	*r*	*p*
TOMM40	−0.737	8.38E-12	−0.801	5.77E-15
ANKH	0.693	4.20E-10	0.770	2.58E-13
DUSP9	−0.689	6.05E-10	−0.723	3.19E-11
MRPS30	0.654	8.15E-09	0.620	7.58E-08
MRAS	−0.637	2.53E-08	−0.703	1.84E-10
LPA	−0.604	1.97E-07	−0.579	8.38E-07
TCF7L2	0.591	4.24E-07	0.583	6.51E-07
CDKN1B	0.578	8.62E-07	0.598	2.84E-07
PPAP2B	−0.563	1.94E-06	−0.633	3.39E-08
GCKR	0.550	3.57E-06	0.582	7.05E-07
MRPS6	−0.545	4.63E-06	−0.573	1.11E-06
SRR	−0.523	1.29E-05	−0.563	1.89E-06
SCD5	−0.426	5.59E-04	−0.497	4.01E-05
PTPRD	0.398	1.36E-03	0.417	7.51E-04
BDNF	−0.353	4.87E-03	−0.427	5.41E-04

*Only significant results are displayed. Results are arranged in order of decreasing *p*-value*.

### SUVR Uptake and Allele Frequencies

There were significant differences in average SUVR for the whole brain as a function of allele frequencies for two genes. TOMM40 A/A allele frequencies were associated with significantly greater SUVR when compared to A/G expression in the right (*p* = 0.002) and left (*p* = 0.001) cortex (Figure 4A). A/A expression was also associated with significantly greater SUVR when compared to G/G expression in the right (*p* = 0.05) and left (*p* = 0.045) cortex. DUSP9 A/G allele frequencies were associated with significantly greater SUVR when compared to A/A expression in the right (*p* = 0.021) and left (*p* = 0.005) cortex (Figure 4B). A/G expression was also associated with significantly greater SUVR when compared to G/G expression in the left cortex (*p* = 0.021).

**Figure 3:**
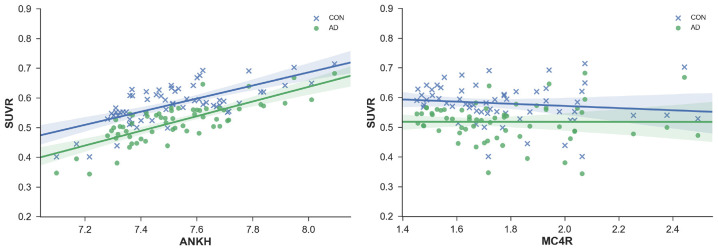
SUVR plotted against regional gene expression for genes with a significant interaction effect. Each point represents a region of the DKT atlas.

**Figure 4:**
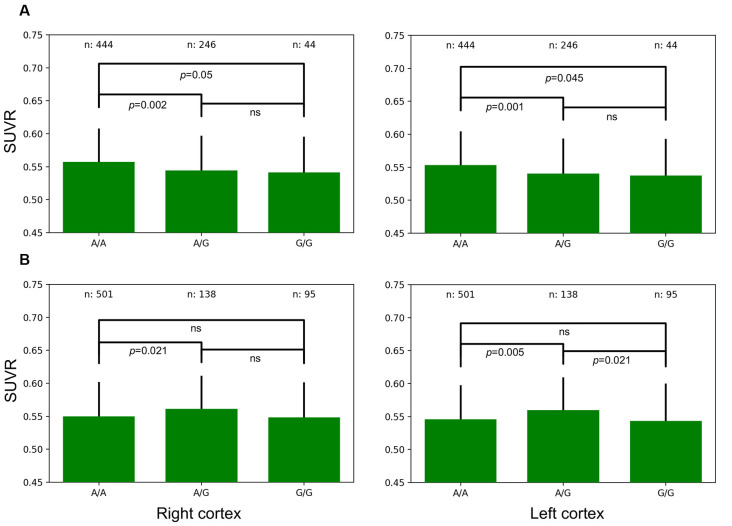
Left and Right whole-brain cortical SUVR expressed as a function of allele frequencies for TOMM40 **(A)** and DUSP9 **(B)**.

## Discussion

Our results highlighted several significant associations between gene expression and SUVR uptake for both the healthy adults and AD groups. TOMM40 expression was most highly associated with SUVR and explained the most variance in the linear regression models for both the healthy adults and AD groups. PPAP2B, PHACTR1, ANKH, PAM, and CDKAL1 were also associated with SUVR.

### TOMM40

GWAS have reported an increased risk for AD associated with the rs2075650 (TOMM40) minor allele (Kulminski et al., 2019). The TOMM40 gene encodes the outer mitochondrial membrane pore subunit (TOM40), a pore that enables the transport of proteins into mitochondria and is necessary for proper mitochondrial functions (Humphries et al., 2005). TOMM40 is protective of mitochondrial function and is necessary for adequate oxidative phosphorylation, deficits of which may result in cognitive difficulties (Zeitlow et al., 2017). Linkage disequilibrium (LD) between rs2075650 (TOMM40) and rs429358 (SNP encoding the APOE e4 allele) are modest, while previously often only been considered in unison with APOE, recent studies have found that TOMM40 may have effects that are independent of APOE (Roses et al., 2016). We found that rs2075650 minor allele frequencies were associated with lower SUVR in the cerebral cortex (Figure 4). TOMM40 may be an important therapeutic target for the treatment of glucose hypometabolism which has long been associated with AD.

### PPAP2B

Schunkert et al. (2011) identified in a GWAS the minor allele C at rs17114036 (PPAP2B) as being associated with coronary artery disease. The PPAP2B gene encodes the enzyme LPP3, which is a glycoprotein that is localized in the cell plasma membrane. LPP3 is blood flow-sensitive and promotes anti-inflammatory responses *via* the inhibition of LPA signaling. LPA exerts growth-factor like effects *via* the stimulation of cell migration. Inactivation of LPP3 or elevated LPA has been associated with vascular dysfunction and is thought to contribute to the pathology of various disorders including cancer and atherosclerosis (Ren et al., 2013; Mega et al., 2015). The vascular burden has been associated with cognitive performance in older adults (Decarli et al., 2019) and is commonly present in dementia patients (Schneider et al., 2007). Interventions aimed at alleviating vascular burden are promising for the treatment of impaired cognition in older adults. Vascular complications have also been highlighted as a substantial burden in T2D patients (Kosiborod et al., 2018).

### PHACTR1

rs12526453 (PHACTR1) has been associated with coronary artery calcification through genome-wide association studies (Van Setten et al., 2013). Increased PHACTR1 expression has been found in atherosclerotic lesions (Reschen et al., 2016). The PHACTR1 gene product has an inhibitory effect on protein phosphatase 1 (PP_1_). Phactr1 protein is selectively expressed in the brain, where high levels were found in the cortex, hippocampus, and striatum, with enrichment of the protein at synapses (Allen et al., 2004). PP_1_ is part of a family of serine/threonine phosphatases that are present in both the nucleus and cytoplasm (Reschen et al., 2016). PP_1_ is involved in a wide array of physiological processes, including muscle contraction, glycogen metabolism, neuronal signaling, and actin cytoskeleton organization (Sagara et al., 2009).

### ANKH

ANKH is a transmembrane protein that exports the enzyme pyrophosphate (PP_i_). TNF-α is responsible for a decrease in ANKH expression and subsequent reduction in PP_i_ export (Bessueille and Magne, 2015). PP_i_ is a mineralization inhibitor with crystal formation resulting from decreased PP_i_ levels. PP_i_ also plays a role in lipid metabolism as well as calcification. Mutations in ANKH result in diseases associated with excessive mineralization, including calcification of arteries leading to atherosclerosis (Bessueille and Magne, 2015).

### PAM

PAM is an enzyme that catalyzes the carboxy-terminal amidation of glycine-extended peptide which is an important step in the posttranslational modification of many bioactive neuropeptides. Amidation is a process that increases the biological potency of a peptide. Schafer et al. (1992) found PAM expressed in all major brain areas of the adult rat except for the cerebellum. The highest levels were found in the hypothalamus, hippocampus, and olfactory cortex. Thomsen et al. (2018) found that PAM silencing leads to decreased insulin exocytosis in response to glucose. Therefore, PAM is a critical element in the mobilization of insulin in response to glucose.

### CDKAL1

A variant of the *CDKAL1* gene was reported to be associated with T2D and reduced insulin release in humans (Stancáková et al., 2008). Ohara-Imaizumi et al. (2010) studied the effects of the CDKAL1 gene using CDKAL1 knockout mice. The authors determined that CDKAL1 controls insulin release by facilitating ATP generation, leading to the activation of ATP-sensitive K^+^ channels and the subsequent increase in Ca^2+^ concentrations. However, while CDKAL played a critical role in insulin release, knockout mice still had normal glucose homeostasis. Therefore, additional genetic and environmental factors may be needed to cause impaired glucose tolerance.

### Interaction Effects

There appears to be an interaction between probable AD patients and healthy adults for SUVR correlations with the genetic expression for ANKH and MC4R (Figure 3). The slope for the positive correlation between ANKH expression and SUVR was greater in probable AD than healthy adults. Increasing MC4R expression was associated with lower SUVR for healthy adults but not for probable AD patients. Mutations in MC4R are associated with hyperphagia, severe childhood obesity, and hyperinsulinemia. MC4R is a key regulator of energy balance, influencing food intake, and energy expenditure through functionally divergent central melanocortin neuronal pathways (Chambers et al., 2008). Alterations in MC4R signaling affect glucose utilization and insulin sensitivity.

### Allelic Expression

**Figure 5:**
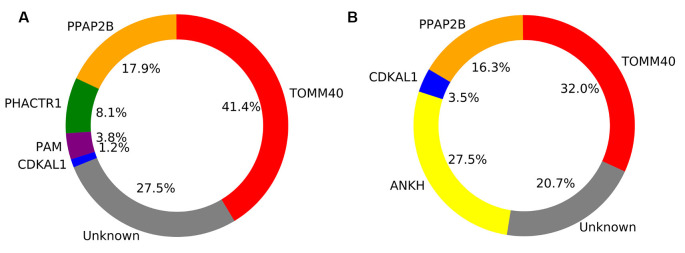
Pie chart showing the percentage of explained variance for each gene retained in the general linear model for Healthy adults **(A)** and Alzheimer’s disease (AD) patients **(B)**.

A/A TOMM40 allelic expression resulted in significantly higher SUVR uptake, while A/G DUSP9 allelic expression resulted in significantly higher SUVR uptake in the right and left cortical hemispheres (Figure 5). Enhanced DUSP9 expression has a protective effect against the development of insulin resistance by counteracting the effects of proinflammatory cytokines, such as TNF-α (Emanuelli et al., 2008). Increasing DUSP9 activity is a potential therapeutic target for the treatment of insulin-resistant disorders.

### Limitations

There are several limitations associated with this study that must be acknowledged. First, our analysis included all AD cases in ADNI regardless of race or ethnicity which may affect FDG-PET brain metabolism. The participants included in the HBA sample were aged 24–57 while the ADNI participants were significantly older. While we assumed gene expression does not change during aging, the possibility of changes in gene expression with age, or in AD, should be further explored when considering any specific gene. Furthermore, given that we analyzed SUVR across several brain regions our results are less affected by isolated changes in gene expression in one region or a set of specific regions. Next, the HBA sample is small, with only six participants. Hence, we operated under the strict assumption that the genetic profile of the HBA would match that of the ADNI participants. There was a very small but significant age difference between the older adults and AD groups. Also, The AD group had significantly lower BMI and a lower proportion of Females. These differences may have affected the results of the study. Future studies should control for these variables. Despite these significant limitations, the uniqueness of the HBA sample makes it indispensable for exploratory studies such as this. We are not aware of any comparable data in a group of older adults that would match the ADNI FDG-PET sample.

## Conclusion

Our results found an association between expression of risk factor genes for T2D and glucose metabolism in older adults and probable AD patients. These genes were associated with mitochondrial stability, vascular maintenance, and glucose intolerance. Future studies should assess non-conventional T2D interventions, including targeting risk-factor genes such as TOMM40, to improve glucose metabolism in older adults and AD patients.

## Data Availability Statement

All datasets presented in this study are included in the article/Supplementary Material.

## Ethics Statement

The studies involving human participants were reviewed and approved by the local ethics board and informed consent of the participants were obtained as part of the ADNI study. The patients/participants provided their written informed consent to participate in this study.

## Author Contributions

SN prepared the manuscript and figures. OP, SC, T-HC, and SD reviewed the manuscript. All authors contributed to the article and approved the submitted version.

## Conflict of Interest

SD is an officer and shareholder of True Positive Medical Devices Inc.

The remaining authors declare that the research was conducted in the absence of any commercial or financial relationships that could be construed as a potential conflict of interest.

## Footnotes

^1^http://www.brain-map.org

^2^http://adni.loni.usc.edu

^3^http://www.adni-info.org

^4^https://github.com/UCL/PETPVC

## Abbreviations

AD: Alzheimer’s disease
ADAS-Cog: AD Assessment Scale-cognitive subscale
ADNI: AD Neuroimaging Initiative
MCI: Mild Cognitive Impairment
MMSE: Mini-Mental State Examination
SNP: Single Nucleotide Polymorphism
T2D: type 2 diabetes.
