# Supplementary material for: Associating Type 2 Diabetes Risk Factor Genes and FDG-PET Brain Metabolism in Normal Aging and Alzheimer’s Disease
Source: Front Aging Neurosci. 2020 Oct 30;12:580633. doi: 10.3389/fnagi.2020.580633 (PMC7661639; doi:10.3389/fnagi.2020.580633)
Supplement: Supplementary file 1 [file Table_1.docx]

Supplemental Table 1

| Nearest gene | SNPs | Donor consistency |
| --- | --- | --- |
| ANKH | rs78408340 | 0.85 |
| MRAS | rs2306374 | 0.73 |
| DUSP9 | rs5945326 | 0.70 |
| PPAP2B | rs17114036 | 0.68 |
| MRPS30 | rs62368490 | 0.67 |
| SRR | rs391300 | 0.66 |
| GCKR | rs1260326 | 0.64 |
| SCD5 | rs12642790 | 0.64 |
| MRPS6 | rs9982601 | 0.63 |
| MC4R | rs571312 | 0.61 |
| CDKN2A | rs10965250 | 0.60 |
| PATJ | rs12140153 | 0.59 |
| CDKN1B | rs2066827 | 0.57 |
| LPA | rs3798220 | 0.56 |
| TMEM163 | rs998451 | 0.56 |
| WDR12 | rs6725887 | 0.55 |
| LDLR | rs1122608 | 0.53 |
| SLC30A8 | rs3802177 | 0.51 |
| BDNF | rs10767664 | 0.50 |
| POC5 | rs2307111 | 0.48 |
| C6orf57 | rs1048886 | 0.47 |
| RREB1 | rs9379084 | 0.45 |
| PCSK9 | rs11206510 | 0.45 |
| PHACTR1 | rs12526453 | 0.44 |
| WSCD2 | rs1426371 | 0.43 |
| CDKN2B | rs10965250 | 0.42 |
| CDKAL1 | rs7766070 | 0.42 |
| SEC16B | rs543874 | 0.42 |
| PPARG | rs13081389 | 0.42 |
| BTN2A1 | rs6929846 | 0.40 |
| TOMM40 | rs2075650 | 0.38 |
| IRS2 | rs4771648 | 0.38 |
| ZNF259 | rs964184 | 0.38 |
| FTO | rs16945088 | 0.37 |
| KCNQ1OT1 | rs2237895 | 0.36 |
| IRX1 | rs11748327 | 0.35 |
| NEUROG3 | rs41277236 | 0.34 |
| CXCL12 | rs1746048 | 0.32 |
| SH2B3 | rs3184504 | 0.31 |
| TCF7L2 | rs7903146 | 0.31 |
| ABO | rs579459 | 0.30 |
| HNF1A | rs1800574 | 0.29 |
| PTPRD | rs17584499 | 0.28 |
| HNF1A | rs56348580 | 0.27 |
| GNPDA2 | rs10938397 | 0.27 |
| QSER1 | rs145678014 | 0.23 |
